# Enzymes as Biomarkers of Environmental Stress in African Catfish (Clarias gariepinus) in Osun State, Nigeria

**DOI:** 10.5696/2156-9614-7.14.71

**Published:** 2017-06-22

**Authors:** Omolara Titilayo Aladesanmi, Femi Kayode Agboola, Rapheal Emuebe Okonji

**Affiliations:** 1 Institute of Ecology and Environmental Studies, Obafemi Awolowo University, Ile-Ife, Nigeria; 2 Department of Biochemistry, Obafemi Awolowo University, Ile-Ife, Nigeria

**Keywords:** enzymes, antioxidant, detoxifying, bioaccumulation, biomarkers

## Abstract

**Background.:**

Many natural aquatic bodies have been contaminated with heavy metals released from domestic, industrial and other anthropogenic activities. Fish are an important bioindicator species and play an important role in the monitoring of water pollution.

**Objectives.:**

This study shows the effect of heavy metals on the distribution of glutathione S-transferases (GST), catalase, rhodanese and 3-mercaptopyruvate sulphur transferase (3-MST) isolated from the liver, gills, fins and muscle of Clarias gariepinus.

**Methods.:**

Glutathione S-transferase, catalase, rhodanese and 3-mercaptopyruvate S-transferase enzymes were isolated from the liver and gills of fish by homogenization of each tissue (with specific buffers for each enzyme) and centrifugation. Serial dilutions of the crude enzymes were then assayed for residual enzymatic activities using standard enzyme assay protocol.

**Results.:**

The results showed heavy metals in the liver and muscle of the investigated fish. This study indicated significant accumulation of heavy metals in the tissues/organ of the fish from Ilesha, Osogbo and Yakoyo fish ponds. These are three main towns in Osun State where the major occupation is fish farming. The relationship between enzymatic activities and heavy metal content in C gariepinus tissue showed positive and significant (p<0.05) correlations between lead (Pb) and GST as well as chromium (Cr) and GST. This implies that higher concentrations of Pb and Cr induced the expression of greater GST activity in the fish tissue.

**Conclusions.:**

The study concluded that the pattern of response of GST, catalase, rhodanese and 3-MST activities in the various organs/tissues of C gariepinus to the heavy metals suggests that the excitation or inhibitions of their activities are organ specific. Further biochemical studies of fish tissues/organs are needed to characterize the enzymatic changes associated with heavy metal pollution.

## Introduction

The contamination of fresh water systems with a wide range of pollutants has become a matter of global concern. Many natural aquatic bodies have been extensively contaminated with heavy metals released from domestic, industrial and other anthropogenic activities. This may have serious effects on the ecological balance of the recipient environment.^1^,^2^ Organisms present in the aquatic environment may accumulate toxic metals, which ultimately affect not only the productivity and reproductive capabilities of the organisms, but also the health of the human beings that depend on the organisms as a major source of protein. As with other aquatic animal species, fish cannot escape the detrimental effects of these pollutants.^3^,^4^ Studies carried out on various fish species have revealed that heavy metals may alter biochemical parameters both in tissues and in blood.^5^,^6^

Fish are an important bioindicator species and play an increasingly important role in the monitoring of water pollution, because they respond with great sensitivity to changes in the aquatic environment.^7–10^ The sudden death of fish can indicate heavy pollution, and the effects of exposure to sub-lethal levels of pollutants can be measured in terms of their biochemical, physiological or histological responses.^11^ The binding of a toxic compound like a heavy metal with its receptor may induce cellular processes that have toxic or other adverse effects on the cell (deoxyribonucleic acid and nuclear protein).^12^,^13^ In macroorganisms, these processes subsequently affect organs, the organism itself or even the whole population. The acute, chronic and long term effects of chemical compounds on living systems can be studied by evaluating the biochemical and morphological changes in various organs, especially the liver of the fish.^14^ The liver is the principal organ of metabolism and plays a role in many body processes, most especially in the detoxification of chemical compounds. Some of the enzymes involved in detoxification reactions in the liver can be used as biomarkers of exposure to toxic chemical pollutants. Biochemical markers are measurable responses of the exposure of an organism to toxic compounds. The biochemical markers measure effects of, or exposure to, toxic chemicals, type of toxicity, and the magnitude of their response often correlates with the level of pollution.^15^,^16^ The use of biomarkers is a promising tool in health risk assessment of exposure to potentially toxic chemicals.^2^

The removal of these toxic compounds and even some endogenous substances from the cell is catalyzed by a number of different enzymes, called phase I and II enzymes. Biotransformation results from the activities of phase I and phase II metabolizing enzymes that are mainly, although not exclusively, located within the hepatocyte.^17^,^18^ Phase I biotransformation generally consists of oxidative metabolism by cytochromes P450. The resulting metabolites are often electrophilic compounds that can bind to cellular proteins or nucleic acids and alter cell functions.^17^,^18^

The antioxidant defense system includes antioxidant enzymes such as catalase (EC 1.11.1.6). This free radical scavenger can eliminate electrophilic chemicals or metabolites and reduce organic peroxides. Increased glutathione S-transferases (GST) activity in fish liver has been demonstrated in various fish species as the result of exposure to polychlorinated biphenyls, polycyclic aromatic hydrocarbons, pesticides and heavy metals.^2^,^19–22^ Glutathione S-transferases (GSTs: EC. 2.5.1.18) are a family of phase II detoxification enzymes and are also known to provide protection against oxidative stress.^2^ Consequently, catalase is the enzyme that breaks hydrogen peroxide (H^2^O^2^) into water and oxygen. H^2^O^2^ is a potent oxidizing agent that can cause damage in a living cell; because of this, any cell that uses oxygen or can live in the presence of oxygen must have a way to get rid of the peroxide. One way is to make use of antioxidant enzymes such as catalase. Other important detoxifying enzymes are rhodanese and 3-mercaptopyruvate which break down toxic compounds into less toxic compounds in a process called detoxification reaction.^2^ Rhodanese (thiosulphate: cyanide sulphurtransferase, EC 2.8.1.1) is a sulphur transferase that catalyses the formation of thiocyanate from free cyanide and a sulphur donor. It is a ubiquitous enzyme that is active in bacteria, yeast, plants, and animals. This enzyme belongs to the family of transferases, specifically the sulfurtransferases, which transfer sulfur-containing groups. The systematic name of this enzyme class is 3-mercaptopyruvate sulphur transferase (3-MST): cyanide sulfurtransferase. This enzyme is also called beta-mercaptopyruvate sulfurtransferase. This enzyme participates in cysteine metabolism.^2^

Therefore, enzymatic parameters are important in diagnosing the functional status of fish exposed to pollutants. It is also important to establish the bioaccumulation of toxic compounds in the tissues of fish in order to determine whether or not they exceed the acceptable guidelines for consumption. This study investigates the changes in enzymatic parameters of the African catfish, Clarias gariepinus, cultivated in three selected ponds receiving water from natural streams in Osun State, Nigeria to determine the concentration of the fish tissues/organs and compare with the standard threshold level, determining whether the fish are suitable for consumption.

**Abbreviations**
*3-MST*: 3-mercaptopyruvate sulphur transferase
*Cd*: Cadmium
*Co*: Cobalt
*Cr*: Chromium
*Cu*: Copper
*Fe*: Iron
*NESREA*: National Environmental Standards and Regulations Enforcement Agency
*GST*: Glutathione S-transferases
*H^2^O^2^*: Hydrogen peroxide
*Mn*: Manganese
*Ni*: Nickel
*Pb*: Lead
*WHO*: World Health Organization
*Zn*: Zinc

## Methods

### Sampling Source

**Figure 1:**
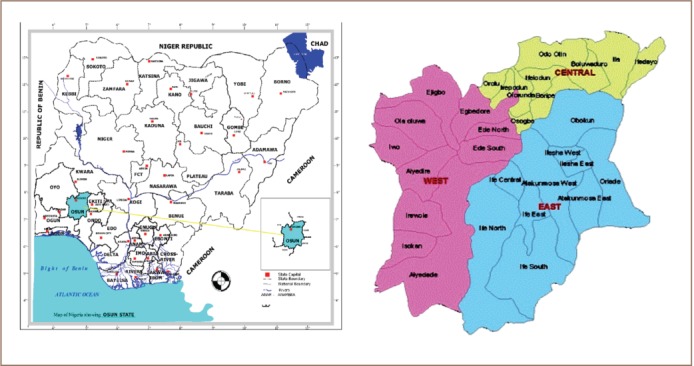
Map of Nigeria showing Osun State and sampling points Source: Google Maps

Clarias gariepinus were collected from selected ponds in Osogbo, Ilesha and Yakoyo areas (Nigeria) of Osun State with the aid of gill nets. They were brought alive to the laboratory in an aerated container containing the stream water, avoiding injury during transportation. Osun State is located between latitude 6.55° and 8.10° North and longitude 3.55° and 5.05° East (Figure 1) and most residents are fishermen by occupation. The selected streams have potential pollution-causing activities close to them. This includes a cement block factory and automobile workshop in Osogbo, dumpsites and improper sewage discharge in Ilesha and a cement block factory in Yakoyo. The fish were disinfected with 0.1% potassium permanganate and then preserved in the freezer at −4°C until use.^23^,^24^ To euthanize the fish, they were placed in the freezer because of the enzyme analyses that were done on the organs.

### Tissue and Organ Collection

Ten adult Clarias gariepinus were weighed, measured, placed on a dissection board and excised to remove the liver, fins, muscles and gills. About 50 g of each tissue type was collected. Samples were kept on ice before being transferred to a deep freezer (−4°C) and kept for further analysis.

### Heavy Metals Analysis

Fish samples were dried at 75°C (to remove all water of inclusion) and blended. Two grams (2.0 g) of the fish tissue samples were ashed separately at 550°C overnight in a furnace. Ashed samples were then transferred quantitatively to 100 ml glass (Pyrex) beakers. Crucibles used for ashing were washed with 25 ml of 20% nitric acid solution as part of qualitative transferring. The washouts were added to the ashed samples in a beaker and then warmed in a fume hood just up to boiling. The solution was left to cool and then filtered using gravity into a 50 ml volumetric flask and made to the mark with distilled water.^25–27^ Samples were run on an atomic absorption spectrophotometer for the presence of lead (Pb), chromium (Cr), zinc (Zn), nickel (Ni), cadmium (Cd), copper (Cu), iron (Fe), cobalt (Co), and manganese (Mn).

### Evaluation of Enzymatic Activity

Glutathione S-transferase, catalase, rhodanese and 3-mercaptopyruvate S-transferase enzymes were isolated from the liver and gills of the fish by homogenization of each tissue (with specific buffers for each enzyme) and centrifugation. Serial dilutions of the crude enzymes were then assayed for residual enzymatic activities in triplicates using various standard methods.

GST activity was determined by the method described by Habig *et al*., using 0.1 M phosphate buffer with pH 6.5.^28^,^29^ This follows the increase in absorbance at 340 nm due to the formation of the conjugate 1-chloro- 2,4-dinitrobenzene used as substrate at the presence of reduced glutathione. Catalase activity was determined by the method described by Xu *et al*.^30^ This method was based on the first-order reaction of catalase with H_2_O_2_. The enzyme sample was added to the H_2_O_2_-phosphate buffer solution and the absorbance of H_2_O_2_ at 240 nm was measured every 5 seconds for 60 seconds by a spectrophotometer.

Rhodanese (a detoxifying enzyme) activity was measured according to the method of Lee *et al*., as modified by a previously described method using a 50 mM borate buffer of pH 9.^31–33^ The absorbance was read at 460 nm against a reagent blank containing no enzyme sample. Mercaptopyruvate sulphurtransferase, another detoxifying enzyme activity, was measured according to the modified method of Taniguichi and Kimura as previously described.^34^,^35^ The reaction mixture consisted of a final concentration of 0.5 ml of 0.38 M Tris buffer pH 7.8, 0.2 ml of 0.5 M potassium cyanide, 0.2 ml of 0.3 M 2-mercaptopyruvate and 0.1 ml of enzyme sample. The absorbance was read at 460 nm against a reagent blank containing no enzyme sample.

### Determination of Protein Concentration

The total protein concentration was determined using the modified method of Bradford by Agboola and Okonji with bovine serum albumin as standard.^36^,^37^ The reaction mixtures were incubated for 15 minutes and the absorbance was read at 595 nm against a reagent blank. A standard curve was obtained from the plot of absorbance versus protein concentration. Protein concentration was then interpolated from a standard curve.

### Statistical Analysis

We analyzed the data using statistical analysis software, SAS/STAT^®^ software version 9.2. The information/data obtained were analyzed using descriptive and inferential statistics. One-way analysis of variance and Duncan multiple range test (for separation of means) were used to evaluate the significant difference in the mean concentration of different studied metals with respect to different samples. A probability level of 0.05 or less was considered significant. Standard errors were also estimated.

## Results

### Bioaccumulation of Heavy Metals in Fish Tissues/Organs

Table 1 shows the results of the bioaccumulation of heavy metals in the tissues of the investigated fish, Clarias gariepinus. Fish liver showed significantly higher levels (p<0.05) of Pb, Fe, Cu, Zn, Cr, Cu, Mn and Ni (Ilesha pond); Cu and Fe (Osogbo pond); as well as Ni, Cu, Fe and Mn (Yakoyo pond). The distribution pattern of most of the heavy metals in Ilesha, Osogbo and Yakoyo was liver>gills>muscle>fins. A general observation of the values of Zn, Fe, Cu, Ni and Mn (30.03±2.08 μg/kg, 133.03±17.09 μg/kg and 62.11±5.85 μg/kg,) recorded in C gariepinus tissues showed that the concentrations were high, relative to other metals (*Table 1*).

**Table 1:** Bio-accumulation Level of Heavy Metals in the Tissues/Organs of C gariepinus from Ilesha, Osogbo and Yakoyo Fish Ponds

	**Heavy Metals (μg/g)**	**Muscle**	**Gills**	**Fins**	**Liver**
**Ilesha**	Pb	53.01^a*^±7.01	3.00^b^±0.04	1.78^b^±0.03	6.01^ab^±4.19
	Cr	13.00^b^±1.02	99.01^a^±10.03	33.01^ab^±3.00	82.32^a^±9.26
	Zn	30.03 ^b^±2.14	45.11^ab^±3.11	60.08^a^±5.14	70.17^a^±6.98
	Ni	27.02^b^±2.01	17.01^b^±1.19	33.02^b^±2.09	573.34^a^±89.53
	Cd	BDL	BDL	BDL	BDL
	Cu	302.02^a^±29.77	446.00^a^±40.04	457.03^a^±43.42	703.01^a^±92.04
	Fe	12.12^b^±0.09	27.13^b^±2.11	10.10^b^±1.06	133.03^a^±10.63
	Co	34.14a^b^±3.24	103.01^a^±12.31	19.11^b^±1.87	44.17^ab^±3.75
	Mn	62.11^b^±7.01	373.12^a^±20.98	746.02^a^±87.04	345.13^a^±30.84
**Osogbo**	Pb	10.11^a^±0.14	11.20^a^±0.76	2.34^b^±0.01	2.11^b^±0.02
	Cr	93.21^a^±9.11	101.01^a^±9.84	42.00^b^±3.09	89.22^a^±8.41
	Zn	33.00^a^±2.76	14.22^b^±0.98	10.18^b^±1.04	11.10^b^±0.09
	Ni	152.21^a^±10.85	47.31^b^±4.01	53.17^b^±4.37	43.34^b^±4.02
	Cd	BDL	BDL	BDL	BDL
	Cu	132.23^a^±11.02	44.00^b^±3.88	57.77^b^±6.01	201.01^a^±18.54
	Fe	333.41^a^±28.66	127.11^b^±11.04	112.12^b^±10.12	410.53^a^±40.04
	Co	114.12^a^±10.01	103.01^a^±10.55	14.41^b^±0.74	83.12^a^±7.07
	Mn	475.23^a^±39.25	94.22^b^±7.22	79.02^b^±6.99	399.51^a^±33.06
**Yakoyo**	Pb	3.41^a^±0.44	1.03^a^±0.01	1.14^a^±0.01	1.34^a^±0.03
	Cr	113.10^a^±10.11	29.51^b^±0.95	7.01^c^±0.76	72.33^a^±6.04
	Zn	76.13^a^±6.11	15.12^b^±0.46	69.13^a^±5.04	72.22^a^±6.09
	Ni	97.32^a^±9.77	7.71^b^±0.68	3.02^b^±0.03	177.13^a^±10.11
	Cd	BDL	BDL	BDL	BDL
	Cu	72.56^a^±7.55	66.10^a^±5.78	11.21^c^±0.44	83.62^a^±7.76
	Fe	253.24^a^±21.04	271.11^a^±19.84	72.12^b^±6.53	310.00^a^±29.04
	Co	111.14^a^±10.32	10.88^b^±0.87	9.01^b^±0.95	97.12^a^±9.09
	Mn	221.71^a^±20.01	73.42^b^±6.56	46.01^b^±4.08	245.11^a^±22.01

Data represent the mean ± SEM (n=5)

^*^Means with the same letter along the same rows are not significantly different at (p≥0.05)

**Abbreviations:** BDL, below detectable limit.

The concentrations of Zn, Fe and Mn recorded in the present study are below the World Health Organization (WHO) recommended level as shown in Table 2.^38^ However, the value of Cu (302.02 μg/kg) in fish from Ilesa was high when compared to WHO and the National Environmental Standards and Regulations Enforcement Agency (NESREA) limits of 100–300 μg/kg. The Pb level in fish from Ilesha (63.31 μg/L, based on Pb content in water) was the highest, however, it fell within the WHO and NESREA limits (Table 2). The Ni levels of 27.02 μg/kg, 152.21 μg/kg and 97.32 μg/kg in fish tissues at Ilesha, Osogbo and Yakoyo ponds, respectively, were all within the acceptable limit of WHO and NESREA for surface water suitable for aquaculture (*Table 2*).

**Table 2:** Comparison of Heavy Metals in Fish Tissue with WHO and NESREA Standards

**Heavy Metal(μg/kg)**	**Ilesha**	**Osogbo**	**Yakoyo**	**WHO^83^**	**NESREA^84^**	**FAO/WHO^69^**
**Pb**	63.31±6.01	10.11±1.21	3.41±0.28	20	20	50
**Cr**	13.10±1.07	93.21±9.07	113.10±10.07	150	150	
**Co**	34.14±2.31	114.12±11.01	111.14±10.11		-	
**Zn**	30.03±2.08	33.00±4.08	76.13±6.76	>10000	75000	40000
**Ni**	27.02±2.01	152.21±13.21	97.32±0.81	600	500	
**Mn**	62.11±5.85	475.23±38.85	221.71±21.85	500	500	
**Fe**	133.03±17.09	333.41±30.12	253.24±27.09		-	
**Cu**	302.02±29.79	132.23±12.77	72.56±6.79	300	100–300	300
**Cd**	BDL	BDL	BDL	-	-	

Data represent the mean ± SEM (n=5)

### Biomonitoring of Ilesha, Osogbo and Yakoyo Fish Pond Water

The results of the distribution of catalase, GST, 3-MST and rhodanese activities in the liver, gills, fins and muscle determined in this study are shown in Tables 3 to 7. Enzyme activities (μmol/min/mg protein) across the tissues/organs of C gariepinus were all significantly different (p<0.05), with liver having the highest level of enzymatic activities and 3-MST showing the highest significant activity (p<0.05) across body tissues (Table 3). Other enzyme (such as catalase, GSTs and rhodanese) activities in the catfish tissues were also significant. The activities of rhodanese were higher in all of the tissues (muscle, gills, fins and liver) than other enzymes (*Table 3*).

**Table 3:** Distribution of Enzyme Activities in (μmol/min/mg protein) Across Tissues of C gariepinus

**Enzymes**	**Muscle (n = 3)**	**Gills (n = 3)**	**Fins (n = 3)**	**Liver (n = 3)**	**P- *value***
**Catalase**	85.00±0.00^a*^	23.00±0.00^c^	44.00±0.00^b^	90.00±0.00^a^	< 0.028
**GSTs**	52.00±0.00^b^	44.00±0.00^c^	21.00±0.00^d^	82.00±0.01^a^	< 0.032
**3-MST**	35.00±0.00^b^	39.00±0.00 ^b^	45.00±0.00^a^	59.00±0.01^a^	< 0.002
**Rhodanese**	144.00±0.03^a^	90.00±0.01^b^	54.00±0.00^c^	160.00±0.00^a^	< 0.051

Values are means ± SEM

^*^Means with the same letter along the same rows are not significantly different at (p≥0.05)

**Abbreviations:** BDL, below detectable limit.

**Table 4:** Effect of Location Variation on Catalase Activity in Different Tissues of C gariepinus (μmol/min/mg protein)

**Location**	**Muscle**	**Gills**	**Fins**	**Liver**
**Ilesha**	92.00±0.02^b*^	38..00±0.01^c^	60.00±0.00^b^	113.00±0.01^a^
**Osogbo**	88.00±0.01^a^	43.00±0.02^b^	32.00±0.00^b^	89.00±0.00^b^
**Yakoyo**	45.00±0.00^b^	66.00±0.00^a^	33.00±0.00^b^	76.00±0.00^b^
**P Value**	<0.02	<0.03	<0.10	<0.03

Values are means ± SEM

^*^Means with the same letter along the same rows are not significantly different at (p≥0.05)

Unit = μmol/min/mg protein.

**Table 5:** Effect of Location Variation on GST Activity in Different Tissues of C gariepinus (μmol/min/mg protein)

**Location**	**Muscle**	**Gills**	**Fins**	**Liver**
**Ilesha**	524.00±0.02^b*^	98.00±0.00^a^	37.00±0.03^a^	138.00±0.01^a^
**Osogbo**	54.00±0.00^b^	68.00±0.01^ab^	21.00±0.01^b^	217.00±0.01^a^
**Yakoyo**	123.00±0.01^a^	42.00±0.00^b^	24.00±0.00^b^	139.00±0.02^a^
**P Value**	<0.041	<0.037	<0.042	<0.192

Values are means ± SEM

^*^Means with the same letter along the same rows are not significantly different at (p≥0.05)

Unit = μmol/min/mg protein.

**Table 6:** Effect of Location Variation on 3-MST Activity in Different Tissues of C gariepinus (μmol/min/mg protein)

**Location**	**Muscle**	**Gills**	**Fins**	**Liver**
**Ilesha**	147.00±0.01^a*^	46.00±0.01^a^	59.00±0.02^a^	105.00±0.01^a^
**Osogbo**	89.00±0.00^a^	36.00±0.00^a^	52.00±0.00^a^	111.00±0.01^a^
**Yakoyo**	92.00±0.02^a^	43.00±0.03^a^	22.00±0.00^a^	102.00±0.01^a^
**P Value**	<0.164	<0.126	<0.118	<0.107

Values are means ± SEM

^*^Means with the same letter along the same rows are not significantly different at (p≥0.05)

Unit = μmol/min/mg protein.

**Table 7:** Effect of Location Variation on Rhodanese Activity in Different Tissues of C gariepinus (μmol/min/mg protein)

**Location**	**Muscle**	**Gills**	**Fins**	**Liver**
**Ilesha**	153.00±0.02^a*^	135.00±0.00^a^	76.00±0.01^a^	252.00±0.03^a^
**Osogbo**	175.00±0.00^a^	79.00±0.05^b^	37.00±0.02^a^	179.00±0.01^a^
**Yakoyo**	151.00±0.04 ^a^	111.00±0.01^ab^	51.00±0.00^a^	222.00±0.00^a^
**P Value**	<0.103	<0.041	<0.141	<0.137

Values are means ± SEM

^*^Means with the same letter along the same rows are not significantly different at (p≥0.05)

Unit = μmol/min/mg protein.

Catalase had the highest activity (113.00 μmol/min/mg protein) in C gariepinus liver from Ilesha, while the lowest catalase activity (32.00 μmol/min/mg protein) was recorded in C gariepinus fins from Osogbo (*Table 4*). However, the lowest catalase activity was not significantly different from what was recorded in Ilesha and Yakoyo fins (*Table 4*).

Similarly, GST activity reached the peak (217.00 μmol/min/mg protein) in Ilesha fish liver and the least activity (21.00 μmol/min/mg protein) was observed in Ilesha fish fins with the two activity peaks showing no significant difference across the three locations *(Table 5).*

The activity of 3-MST was highest (147.00 μmol/min/mg protein) in the muscle, while the lowest 3-MST activity (22.00 μmol/min/mg protein) was recorded in Yakoyo fins, with no significant difference observed in fish from these different locations *(Table 6).*

Rhodanese activity, on the other hand reached its peak (252.00 μmol/min/mg protein) in Osogbo liver and the lowest activity (37.00 μmol/min/mg protein) in Ilesha fins *(Table 7).* From the general analysis, liver recorded the highest activity for all investigated enzymes (except 3-MST).

Analysis of the correlation coefficients of enzymatic activities and heavy metal content in C gariepinus tissue is shown in Table 8. The correlation of Pb and GST, as well as that of Cr with GST, were observed to be positive and significant (p<0.05). Cu and Cr also had a significant (p<0.05) positive correlation with Cr, while Zn had a significant (p<0.05) negative correlation with Cr. Cu and Zn showed a significant (p<0.05) positive correlation with Fe, while Zn showed a significant (p<0.05) positive correlation with Mn.

**Table 8:** Correlation between Enzymatic Activity of Fish and Heavy Metal Concentration

	**Catalase**	**GST**	**3-MST**	**RHOD**	**Pb**	**Cr**	**Co**	**Mn**	**Fe**	**Cu**	**Ni**	**Zn**	**Cd**
**Catalase**													
**GST**	0.41												
**3-MST**	−0.31	−0.06											
**RHOD**	0.56	0.28	−0.57										
**Pb**	0.28	0.77^*^	−0.86^**^	0.70^*^									
**Cr**	0.11	0.76^*^	−0.96^***^	−0.50	−								
**Co**	−0.23	−0.11	0.02	−0.19	−0.31	0.86^**^							
**Mn**	−0.65	0.36	0.25	−0.47	−0.37	0.60	-						
**Fe**	−0.28	0.48	−0.49	0.11	0.39	−0.41	−0.41	0.82^*^					
**Cu**	−0.29	0.16	−0.60	−0.24	0.65	0.92***	−0.30	−0.18	0.82^*^				
**NI**	0.66	0.32	0.21	−0.06	−0.31	0.003	−0.20	−0.61	0.59	−0.30			
**Zn**	−0.29	0.36	−0.42	0.06	0.30	−0.80^**^	−0.40	0.89^**^	0.99^***^	0.51	−0.40		
**Cd**	ND	ND	ND	ND	ND	ND	ND	ND	ND	ND	ND	ND	

^*^, ^**^, ^***^ Significant at 0.05, 0.01 and 0.001 respectively.

**Abbreviations:** ND, not detected; RHOD, rhodanese.

## Discussion

### Bioaccumulation of Heavy Metals in Fish Tissues/Organs

Metal bioaccumulation is the process through which an organism concentrates metals in its body from the surrounding medium or food, either by absorption or ingestion.^39^,^40^

Biomonitoring of hazardous substances in tissues of aquatic organisms has been successfully applied recently for heavy metals pollution.^41^ Fish are often at the top of the aquatic food chain and they accumulate metals in concentrations many times higher than that present either in water and/or sediment. Fish can absorb heavy metals through the epithelial or mucosal surface of their skin, gills and gastrointestinal tract.^42^

Fish can regulate metal concentrations to a certain limit, after which bioaccumulation occurs.^43^ The dominance of heavy metals in the liver and muscle of the investigated fish Clarias gariepinus was observed in this study as shown in Table 1. This study clearly indicated significant accumulation of heavy metals in the tissues/organ of the fish from Ilesha, Osogbo and Yakoyo fish ponds. It also supports the results of previous studies that found that heavy metals were more concentrated in the liver than other parts of the fish tissues/organs.^44^ The presence of these metals in the liver suggests that food was probably the major source of metal uptake, rather than absorption through gills and skin.^45^ It has been reported that enhanced metal levels in fish tissues arise through biomagnification at each trophic level and that omnivorous bottom feeders concentrate higher metal levels.^39^C gariepinus is a known voracious bottom feeder and could thus have bioaccumulated high metal levels from the pond sediment. Thus, heavy metal bioaccumulation in liver may result from the liver being the organ responsible for controlling the toxicity of heavy metals. Similar results have been obtained by other authors.^44^,^46^ The results also agree with those of a previous study which determined that Fe, Cu, Zn and Pb concentrations in the liver of carp and mullet were higher than those in fish muscles.^47^,^48^

Since the liver is a target organ of accumulation for many metals, it is often considered a good monitor of water pollution with heavy metals since their concentrations have proportional relevance to those present in the environment.^49^,^50^ The distribution pattern of most of the heavy metals in Ilesha, Osogbo and Yakoyo of liver>gills>muscle>fins observed in this study agrees with the findings of previous studies, showing that muscle is less active than the liver in accumulating heavy metals.^1^,^50–52^ The high metal concentrations in the gills could be due to the metal complexing with the mucus that is impossible to remove completely from the lamellae before analysis.^43^

The higher levels of Fe and Zn in the fish tissue relative to other metals observed in this study could be due to the fact that these metals are naturally abundant in Nigerian soil which is the main source of metals in the surrounding water of the fish samples.^53^,^54^ The soils in the study area belong to the highly ferruginous tropical red soils associated with basement complex rocks.^55^ High concentrations of Cu and Zn in fish were found to mainly bioaccumulate in the skin, liver, gill, heart, kidney and muscle tissue of fish.^56^ It has also been observed that Fe is the dominant metal in the muscle of C gariepinus.^57^

The accumulation pattern in this study revealed that the accumulation of the essential elements Zn, Cu, Fe and Mn were higher than those of the non-essential metals Pb, Co and Cr in the fish tissues in the three locations as previously reported.^58^ The essential elements play vital biochemical and physiological functions in fish. Zn, for instance, is regulated to maintain a certain homeostatic status in fish, while both Fe and Cu are components of the enzyme cytochrome oxidase which is involved in energy metabolism.^58^,^59^ Heavy metals in excess of the body needs of fish or humans may constitute a major pollution source and pose a serious health risk.^60^ The toxicity of Fe may lead to hemochromatosis and, in severe cases, to thalassemia, while excessive intake of Zn may lead to diarrhea and vomiting in humans.^61^ In humans, the toxicity of Mn leads to a syndrome called manganism, which involves both psychiatric symptoms and features of Parkinson disease.^62^

It is noteworthy that the values of Zn, Fe and Mn recorded in the present study are below the WHO recommended level as shown in Table 2.^38^ Consequently, the consumption of C gariepinus from the investigated waterbodies and very likely others in the same catchment basin may not pose any heavy metal (Zn, Fe and Mn) induced health hazards. However, the value of Cu (302.02 μg/kg) was high when compared to WHO and NESREA limits of 100–300 μg/kg in fish at Ilesha. Cu is commonly a natural element in water and sediment. It is insoluble in water, but many of its salts are highly soluble. Cu is a fundamental micronutrient to all forms of life, in enzyme activity and random rearrangement of natural proteins.^63^ At slightly higher but sublethal concentrations, it causes chronic toxicity to aquatic life. Exposure of fish to sublethal concentrations of Cu leads to cardiac activity and reduction in heart rate.^64^ In view of the higher levels of Cu in Ilesha, when compared to WHO and NESREA recommended limits, it could be inferred that consumption of these fish could lead to excess Cu-induced health hazards in humans. Cr levels in Osogbo and Yakoyo fell within the recommended levels.^38^,^65^

The Pb level in fish from Ilesha (63.31 μg/L, based on Pb content in water) was the highest, however, it fell within the WHO and NESREA limits *(Table 2).* Pb is a non-essential element and high concentrations can occur in aquatic organisms close to anthropogenic sources. It is toxic even at low concentrations and has no known function in biochemical processes. It is known to inhibit active transport mechanisms, involving adenosine triphosphate to depress cellular oxidation reduction reactions and to inhibit protein synthesis.^66^ Pb was also found to inhibit impulse conductivity by inhibiting the activities of monoaminooxidase and acetylcholine esterase, to cause pathological changes in tissue and organs and to impair the embryonic and larval development of fish species.^67^ Chronic Pb toxicity in fish leads to nervous damage which can be determined by the blackening of the fins.^68^ Acute toxicity, on the other hand, causes gill damage and suffocation.^69^ Children are more susceptible to Pb poisoning, resulting in clinically overt encephalopathy.

Ni is only moderately toxic to fish and has little capacity for bioaccumulation. Its dominant form in water is Ni^2+^. It enters the aquatic environment through the disposal of batteries and effluents from metal plating and ore processing industries. In humans, nickel can be carcinogenic and teratogenic.^68^ The present study recorded Ni levels of 27.02 μg/kg, 152.21 μg/kg and 97.32 μg/kg in fish tissues at Ilesha, Osogbo and Yakoyo ponds, respectively, which fall within the acceptable limits of the WHO and NESREA for surface water suitable for aquaculture.

### Biomonitoring of Ilesha, Osogbo and Yakoyo Fish Pond Water

Methods of biochemical monitoring (biomonitoring) for exposure to environmental contaminants are of great potential use. Ecological and biological factors should be taken into account to explain variations in enzymatic biotransformation activities in fish. The activities around the investigated streams and the associated fish ponds in Ilesha, Osogbo and Yakoyo are likely to enhance environmental pollution. These activities include the use of fertilizers and other chemicals by farmers, leachate discharge from dumpsites, effluent from factories, and poor sewage disposal by community dwellers. C gariepinus can ingest a wide variety of natural food organisms, including plankton, succulent green leaves, benthic organisms, aquatic invertebrates, larval fish and decomposing organic matter.

Enzymes used as biochemical markers (biomarkers) in this study are antioxidants (catalase and GST) and detoxifying enzymes (rhodanese and 3-MST). Oxidative damage may be minimized by antioxidant defense mechanisms that protect the cell against cellular oxidant and repair systems, which prevent the accumulation of oxidative-damaged molecules.^70^,^71^ Any change in the antioxidative parameters in the metabolic pathways of fish reflected by environmental stress causes significant ecophysiological responses.^72^ The distribution of catalase, GST, 3-MST and rhodanese activities in the liver, gills, fins and muscle were determined in this study *(Tables 4 to 7).* The three enzymes 3-MST, rhodanese and GST are sulphurtranferases and are widely distributed enzymes of prokaryotes and eukaryotes which play a part in the management of the cytotoxicity of reactive oxygen species in aerobic tissues.^73–75^ The presence of these sulphurtranferases (GST, rhodanese and 3-MST) and catalase in the tissues of C gariepinus from Ilesha, Osogbo and Yakoyo is an indication of a strong detoxifying mechanism, a protective and possible physiological mechanism for the survival of these organisms in their environment.^35^ On the other hand, rhodanese acts as a general transferase for the exchange of divalent sulphur in the biosphere.^76^

The liver was observed to have the highest level of enzymatic activity, similar to the results of a previous report, and this is probably due to the liver being the organ responsible for controlling the toxicity of heavy metals and other exogenous compounds.^49^,^50^ The distributions of catalase and GST in this study were similar to the trends observed in a previous study by Yilmaz, who reported the highest enzyme activities in the liver and the lowest in the fins.^50^ The distribution of catalase and GST were similar to the trends observed in previous studies which reported the highest enzyme activities in the liver and the lowest in the fins.^50^ The liver recorded the highest activity for all investigated enzymes (except 3-MST) and also showed higher GST activity related to pollutants.^19^,^77–79^ However, there was variation in the levels of enzymatic activity in the fish across the three water bodies. This variation could be the result of fish sensitivity to the presence of different levels of pollutants in the environment.^80^

Studies of the interrelationship among different variables of the ecosystem are a helpful tool in promoting research and extending the frontiers of knowledge. The study of correlation reduces the range of uncertainty associated with decision making.^81^ Analysis of the correlation coefficients of enzymatic activities and heavy metal content in C gariepinus tissue shown in Table 8 indicated positive and negative relationships between the variables, however, only a few of these relationships were significant (p<0.05). These relationships included between enzyme and enzyme, enzyme and heavy metal or heavy metal and heavy metal. The positive correlation of Pb and Cr implies that higher concentrations of Pb and Cr induced the expression of higher GST activity in the fish tissue, and the higher the concentration of Pb and Cr in the fish tissue, the greater the level of GST activity expressed in the fish tissue. The presence of GST activity inhibitors could play a significant role in the biochemical responses of aquatic organisms.^82^ On the other hand, the negative correlation of Pb and Cr with 3-MST reveals the negative effect of Pb and Cr on the activity of 3-MST. The interrelationships among the essential metals observed in this study demonstrate their dependency.

## Conclusions

Results from this study revealed that the response patterns of GST, catalase, rhodanese and 3-MST activities in the various organs/tissues of C gariepinus to the heavy metals suggest that the excitation or inhibitions of their activities are organ specific. It is therefore recommended that further biochemical examinations of fish tissues/organs are needed to characterize the enzymatic changes associated with heavy metal pollution.

The heavy metals of interest found in measurable quantities were still within safe limits for consumption. Efforts should be concentrated on ensuring that these concentrations are not exceeded. The elements Zn, Fe, Cu, Cr and Mn are essential in the human diet and all play significant roles in metabolic processes. In view of the importance of fish in the human diet, regular biological monitoring of the water and fish meant for consumption should be performed to ensure continuous food safety.
